# Orexinergic Neurotransmission in Temperature Responses to Methamphetamine and Stress: Mathematical Modeling as a Data Assimilation Approach

**DOI:** 10.1371/journal.pone.0126719

**Published:** 2015-05-20

**Authors:** Abolhassan Behrouzvaziri, Daniel Fu, Patrick Tan, Yeonjoo Yoo, Maria V. Zaretskaia, Daniel E. Rusyniak, Yaroslav I. Molkov, Dmitry V. Zaretsky

**Affiliations:** 1 Department of Mathematical Sciences, Indiana University—Purdue University Indianapolis, Indianapolis, IN 46202, United States of America; 2 Park Tudor School, Indianapolis, IN 46240, United States of America; 3 Carmel High School, Carmel, IN 46032, United States of America; 4 Department of Emergency Medicine, Indiana University School of Medicine, Indianapolis, IN 46202, United States of America; Georgia State University, UNITED STATES

## Abstract

**Experimental Data:**

Orexinergic neurotransmission is involved in mediating temperature responses to methamphetamine (Meth). In experiments in rats, SB-334867 (SB), an antagonist of orexin receptors (OX1R), at a dose of 10 mg/kg decreases late temperature responses (t>60 min) to an intermediate dose of Meth (5 mg/kg). A higher dose of SB (30 mg/kg) attenuates temperature responses to low dose (1 mg/kg) of Meth and to stress. In contrast, it significantly exaggerates early responses (t<60 min) to intermediate and high doses (5 and 10 mg/kg) of Meth. As pretreatment with SB also inhibits temperature response to the stress of injection, traditional statistical analysis of temperature responses is difficult.

**Mathematical Modeling:**

We have developed a mathematical model that explains the complexity of temperature responses to Meth as the interplay between excitatory and inhibitory nodes. We have extended the developed model to include the stress of manipulations and the effects of SB. Stress is synergistic with Meth on the action on excitatory node. Orexin receptors mediate an activation of on both excitatory and inhibitory nodes by low doses of Meth, but not on the node activated by high doses (*HD*). Exaggeration of early responses to high doses of Meth involves disinhibition: low dose of SB decreases tonic inhibition of *HD* and lowers the activation threshold, while the higher dose suppresses the inhibitory component. Using a modeling approach to data assimilation appears efficient in separating individual components of complex response with statistical analysis unachievable by traditional data processing methods.

## Introduction

Amphetamine and its derivatives are among the most prevalent abused drugs in the world [1]. Amphetamines can cause various medical complications including myocardial infarctions, ischemic and hemorrhagic strokes, rhabdomyolysis, renal failure, and fatal hyperthermia [2–8]. Despite this, there is little understanding of neural mechanisms underlying hyperthermia produced by amphetamines. For instance a similar dose of amphetamine may cause a mild effect in some patients but be fatal to others; this can occur even though the blood levels of the drug appear low [9, 10].

In rats, amphetamines cause the central release of monoamines and the subsequent activation of the sympathetic nervous system causing in heat generation by brown adipose tissue (BAT) [11, 12] and skeletal muscle [13]. In addition, amphetamines decrease heat dissipation through cutaneous vasoconstriction [14, 15]. The brain areas mediating these effects are not known but we have previously shown that sympathetic and behavioral responses mediated, by the substituted amphetamine MDMA, can be prevented by suppressing neuronal activity in the dorsomedial hypothalamus [16], the area which is now well-established to participate in the control of heat production in the BAT and heat dissipation through the skin [17]. This is one of the few brain regions containing neurons that project trans-synaptically to both the adrenal gland and skeletal muscle [18] and these dual-projecting neurons contain the peptide orexin [19]. It has also been further shown that Meth and d-amphetamine activate orexin-containing neurons [20, 21].

Central activation of orexin receptors increases body temperature, BAT sympathetic nerve activity and thermogenesis, plasma epinephrine, and heart rate [22–26]. The systemic administration of SB-334867 (SB), an OX_1_R antagonist, hereinafter referred as SB, prevents increases in heart rate and mean arterial pressure in models of stress and panic [27, 28], and suppresses hyperthermia caused by stress of injection [29]. Pretreatment with SB has also been shown to significantly attenuate hyperthermia evoked by a moderate but not a low or high dose of Meth [29]. Unfortunately, in the cited study temperature fluctuations resulting from the stress of two intraperitoneal injections (SB and Meth) made it impossible to quantify the effect of SB on low doses of Meth using a traditional data analysis.

The differential effects of SB on the temperature responses to different doses of Meth do not allow for a straight-forward interpretation. Recently, we developed a mathematical model [30] that reproduced dose-dependent temperature responses to methamphetamine. The model described interactions of putative brain nuclei that participate in thermoregulation. We use this model to provide mechanistic explanation of how the orexin antagonist SB-334867 affects complex Meth-induced dynamics of the body temperature.

## Materials and Methods

### Animal experiments

#### Animals

Male Sprague-Dawley rats (280–350 g) were individually housed with a 12 h light cycle at a room temperature of 23–25°C with free access to food and water. All animals for which data are reported remained in good health throughout the course of surgical procedures and experimental protocols as assessed by appearance, behavior, and maintenance of body weight. All procedures described here were approved by the Indiana University School of Medicine Institutional Animal Care and Use Committee and followed NIH guidelines.

#### Surgical Procedures

For measurements of core temperature, rats were implanted with telemetric transmitters (C50-PXT, Data Sciences Int., St.Paul, MN) under isoflurane anesthesia as previously described [31]. The body of the transmitter was placed into the abdominal cavity and sutured to the abdominal wall. After at least seven days of recovery, rats, still in their home cages, were brought to experimental room. Cages were placed on telemetric receivers (RPC-1), and animals were given at least two hours to adapt to the new environment.

#### Drugs

The orexin-1 receptor antagonist SB-334867 (Tocris Bioscience, Ellisville, MO) was dissolved immediately before injection, and administered in the volume of 1 ml/kg. For injections of 10 mg/kg, 10 mg of SB was dissolved in 40 μl DMSO, followed by addition of 60 μl of 1 M HCl, and 900 μl of 10% 2-hydroxypropyl-β-cyclodextrin (Sigma-Aldrich, St. Louis, MO) in saline. For injections of 30 mg/kg, 50 mg of SB was dissolved in 300 μl DMSO, followed by addition of 160 μl of 1 M HCl, and 1200 μl of 10% 2-hydroxypropyl-β-cyclodextrin in saline. These procedures provided clear yellow solutions without evidence of flocculation. The vehicles were prepared in the same way without the drug.

Methamphetamine hydrochloride (Sigma-Aldrich, St. Louis, MO) was dissolved in sterile saline at the time of injections and injected at a volume of 1 ml/kg.

#### Experimental protocol: The effect of SB-334867 on methamphetamine-evoked responses

Two separate experimental series were performed. In the first series SB was administered in the dose of 10 mg/kg. In second series, SB was administered in the dose 30 mg/kg. In each study we have used the corresponding vehicles (see above).

In each series rats were randomly assigned to one of eight groups (n = 6–8 per group): one of two pretreatments (SB vs vehicle), followed by one of four treatments (saline or one of three doses of Meth). Each rat received only one treatment. Experiments were performed in a temperature controlled room at T = 25°C and relative humidity 30–70%. Air temperature and humidity were monitored by independent sensors. For all experiments deviations of the temperature from 25°C did not exceed 0.8°C. After baseline physiological parameters were recorded for at least 30 min, animals received an intraperitoneal injection of either vehicle or SB-334867. Thirty min later, rats received an intraperitoneal injection of either saline or one of three doses of methamphetamine (1 mg/kg, 5 mg/kg, or 10 mg/kg). Although there is a wide range of methamphetamine doses that humans abuse, the doses we chose include ones that are commonly used recreationally (1 mg/kg) as well as larger doses that may be reached by binge users (5 and 10 mg/kg) [32]. For convenience we refer to these doses as “low”, “moderate”, and “high” respectively. Each rat was used for only one injection of Meth or saline.

#### Statistical analysis

The results are presented as the mean ± SEM. Results were compared using a one way ANOVA with repeated measures followed by a Fisher’s LSD post hoc test, where appropriate. A value of P<0.05 was considered to indicate a significant difference in all comparisons. Baseline levels of locomotor activity, temperature, HR and MBP did not differ between groups across the series of experiments, so changes from baseline were analyzed.

### Mathematical Modeling

#### General approach

**Fig 1:**
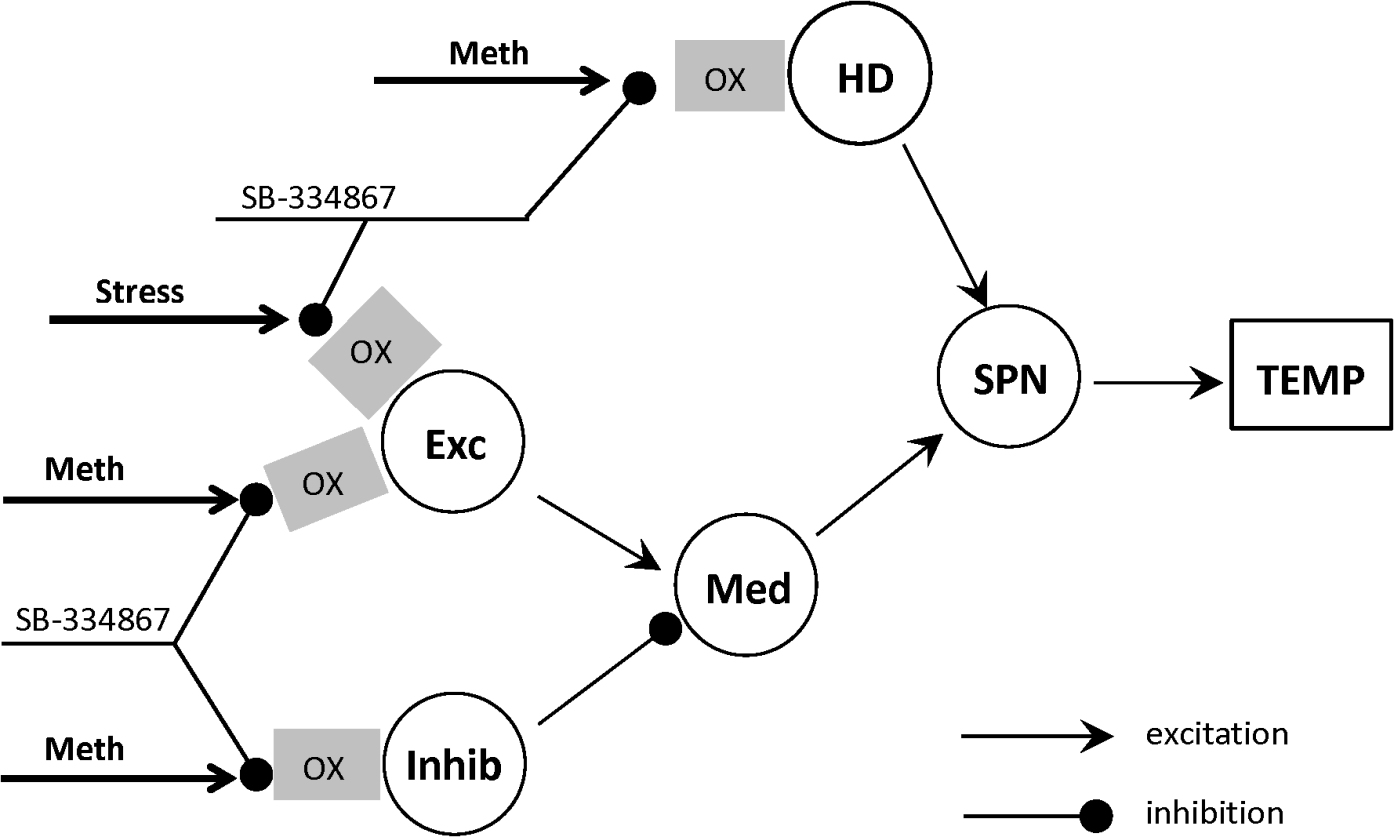
Schematic of the model neuronal circuitry involved in responses to methamphetamine. Stress input and the orexin receptors (shown by gray rectangles labeled “OX”) are added to extend the circuitry used in [30]. Effect of Stress and/or Meth can be affected by the orexin receptor antagonist SB-334867. Each circle represents a neural population. Abbreviations: *Exc*—excitatory node, *Inhib*—inhibitory node, *HD*—high-dose activated node, *Med*—medullary node, *SPN*—sympathetic preganglionic node, *TEMP*—temperature. See text for a detailed description.

In our initial studies [30] we were able to fit experimental data on dose-dependence of temperature responses to Meth with appropriate precision using a mathematical model which was constructed using known anatomy of circuitry involved into responses to amphetamines. The core of the model is a feed-forward sequence of connected excitatory nodes: Excitatory (*Exc*, putatively hypothalamic), Medullary (*Med*) and sympathetic premotor node (*SPN*), out of which *SPN* directly defines a heat generation output (Fig 1). We assume that the signal from SPN is transmitted “as is” to the effector organs controlling thermogenesis. This allows combining the whole downstream chain into a single output. Excitatory node is sensitive to Meth as well as there are two more Meth-sensitive nodes—Inhibitory (*Inhib*) and “high dose” (*HD)*. The Inhibitory drive competes with the Excitatory input to affect the Medullary node, while the *HD* node requires high dose of Meth to be activated, hence the name. Complex dose-dependence of temperature responses to Meth is formed by the competition of two excitatory inputs and one inhibitory input, all of which have different sensitivities to Meth.

Low doses of Meth (1 mg/kg i.p. and below) activate the Excitatory node almost exclusively, and administration of those doses evokes a monophasic mild hyperthermia. Higher doses (3–5 mg/kg i.p.) are able to activate the inhibitory node, which completely masks the activity of the Excitatory node. As a result, immediately after injection of Meth there is no temperature response, but after partial elimination of the drug, the excitatory drive is disinhibited, and temperature increases display a delayed monophasic response. Finally, high doses (10 mg/kg i.p.) activate the *HD* node, and cause immediate powerful hyperthermia followed by a secondary temperature increase due to transient activation of the Excitatory node.

To analyze the orexin data we first modified the existing model to accommodate stress. Since inhibition of the dorsomedial hypothalamus, a putative excitatory node, suppresses sympathetic activation from both stress [33] and amphetamines [16], we hypothesized that stress and Meth both provide direct inputs to the Excitatory node. Each stress stimulus was modeled as a spike of activity of short duration.

Next, we assumed that SB affects Meth and stress inputs at the relevant nodes. Excitatory node exhibits some tonic activity without Meth [30]. If the projections from Excitatory and/or Inhibitory node to Medullary node are orexinergic, the antagonist would cause dramatic (up to 3°C) hypothermia by itself. However, as it will be shown in the Results, the administration of the antagonist alone did not result in any hypothermia whatsoever. Therefore, we were restricted to placing orexin receptors on the Meth-sensitive populations to mediate effects of Meth, but not to cause any perturbations by the receptor blockade alone.

By fitting the temperature responses to Meth after different doses of SB we calculated values of the model parameters.

#### Implementation of the model

The model consisted of three components: pharmacokinetics describing concentration of Meth in the blood after injection; a neural network whose activity was dependent on Meth and SB; and a temperature control system driven by a signal from the neural network.

We assumed that after the injection the drug is being absorbed in the blood from the peritoneum and is also being eliminated from the blood. Accordingly, drug concentrations are described by the following equations:

$$\frac{d\left[M_{p}\right]}{dt}=-\frac{\left[M_{p}\right]}{\tau_{u}}$$ (1)

$$\frac{d\left[M\right]}{dt}=\frac{\left[M_{p}\right]}{\tau_{u}}-\frac{\left[M\right]}{\tau_{d}}$$

where *t* is time in minutes, *M*_*p*_ is the intra-peritoneal drug concentration, and *M* is the blood drug concentration (both in mg/kg). [*M*_*p*_]/*τ*_*u*_ represents drug absorption with time constant *τ*_*u*_, and [*M*]/*τ*_*d*_ represents drug elimination with time constant *τ*_*d*_. Initial conditions are [*M*_*p*_](0) = *D*, [*M*](0) = 0 for injections made at time *0* where *D* is the initial dose of the drug being modeled. System (1) can be solved explicitly as:

$$\left[M\right]\left(t\right)=D{\left(\frac{\tau_{u}}{\tau_{d}}-1\right)}^{-1}\left(e^{-t/\tau_{u}}-e^{-t/\tau_{d}}\right)$$ (2)

for *t* > 0, and [*M*](*t*) = 0 if *t* < 0. Stress input from the injection made at *t* = *t*_0_ was modeled as a single exponential function

$$S\left(t,t_{0}\right)=\{\begin{array}{c} e^{-\left(t-t_{0}\right)/\tau_{s}},\;\text{if}\;t>t_{0}\\ 0,\;\text{if}\;t\leq t_{0} \end{array}$$ (3)

In our study, the neural circuitry is modeled as a feed-forward artificial neural network. The excitatory node *Exc* receives two stress inputs at *t* = −30 min and *t* = 0 min from injections of SB (or vehicle) and Meth (or saline) respectively. The outputs of Meth-sensitive neural populations are calculated as follows:

$$\begin{array}{c} P_{Exc}\left(t\right)=\sigma \left(w_{S}S\left(t,\;-30\right)+w_{S}S\left(t,\;0\right)+w_{Exc}\left[M\right]\left(t\right)+\gamma_{Exc}\right)\\ P_{Inh}\left(t\right)=\sigma \left(w_{Inh}\left[M\right]\left(t\right)+\gamma_{Inh}\right)\\ P_{HD}\left(t\right)=\sigma \left(w_{HD}\left[M\right]\left(t\right)+\gamma_{HD}\right) \end{array}$$ (4)

where *σ*(*x*) = (1 + tanh*x*)/2 is a sigmoid activation function, *w*_*i*_(*t*) is the sensitivity of *P*_*i*_(*t*) to Meth, and *γ*_*i*_ is the basal excitability of *P*_*i*_, *i* ∊ {*Exc*, *Inh*, *HD*}. To account for the effect of the SB, we used different values of (*w*_*S*_, *w*_*Exc*_, *w*_*Inh*_, *w*_*HD*_) for different doses of SB.

The activity of medullary population *Med* is described as follows:

$$P_{Med}\left(t\right)=w_{Exc\to Med}P_{Exc}\left(t\right)-w_{Inh\to Med}P_{Inh}\left(t\right)$$ (5)

where *w*_*Exc*→*Med*_ and *w*_*Inh*→*Med*_ are the weights of the excitatory and inhibitory projections from *Exc* to *Med* and *Inhib* to *Med*, respectively. Inframedullary population *SPN* transmits the activity of *Med* but is additionally excited by the Meth-sensitive *HD* population as follows:

$$P_{SPN}\left(t\right)=P_{Med}\left(t\right)+w_{HD\to SPN}P_{HD}\left(t\right)+\gamma_{SPN}$$ (6)

where *w*_*HD*→*SPN*_ is the weight of the excitatory projection from *HD* to *SPN*, and *γ*_*SPN*_ is the basal excitability of *SPN*.

The temperature response is modeled by a first-order linear ODE driven by the *SPN* signal as follows:

$$\tau_{T}\frac{dT}{dt}=P_{SPN}\left(t\right)-\left(T-T_{0}\right)$$ (7)

where *T* is the body temperature in degrees Celsius, *τ*_*T*_ is the time constant of the temperature response, and *T*_0_ is the baseline body temperature.

#### Statistical Analysis

We used the Bayesian approach for inferring the model parameters. Then, statistical analysis is used to estimate statistical significance of changes in (*w*_*S*_, *w*_*Exc*_, *w*_*Inh*_, *w*_*HD*_) individually when 30, 10, and 0 mg/kg of SB are injected, respectively. To do this, we constructed a corresponding posterior probability density function (PDF) of the parameter vector = (*w*_*S*_, *w*_*Exc*_, *w*_*Inh*_, *w*_*HD*_) considering other parameters fixed at values shown in the Table 1. According to Bayes' rule, this probability distribution is proportional to the probability that an observed temperature time series {*T*_*i*_} is produced by our model with the given ***W***, usually referred to as a likelihood. Assuming that the residuals are normally distributed, the posterior PDF *p*(***W*** | {*T*_*i*_}) is given by the following formula:

$$p\left(W\;|\;\left\{T_{i}\right\}\right)=\exp\left\{-\sum_{\text{i}=-50}^{180}\;\frac{{\left[T_{i}-T\left(W,i\right)\right]}^{2}}{2\sigma_{i}^{2}}\right\}$$ (8)

where *T*(***W***, *i*) is the temperature calculated by our model at time *i* with the given ***W***. *T*_*i*_ and *σ*_*i*_ are respectively a mean and a standard deviation of the body temperature over a corresponding group of animals at time *i* by increments of 2 minutes.

**Table 1:** Values of parameters of the model, which were considered not affected by SB (adapted from [30]).

Parameter	Value	Parameter	Value
***τ*_*u*_(*Meth*), min**	8.25	***τ***_***T***_ **, min**	89.2
***τ*_*d*_(*Meth*), min**	57.5	***T***_**0**_, °*C*	37
***τ*(*Stress*), min**	10	**γ**_**Exc**_ **, *mg/kg***	−0.357
***w*_*Exc*→*Mdl*_, °*C***	9.89	**γ**_**Inh**_ **, *mg/kg***	−1.335
***w*_*Inh*→*Mdl*_, °*C***	6.38	**γ**_**HD**_ **, *mg/kg***	−3.69
***w*_*HD*→*SPN*_, °*C***	5.66	**γ**_**SPN**_ **, °*C***	−3.35

We used the Markov Chain Monte Carlo (MCMC) approach, in particular the modified Metropolis-Hastings algorithm [34–37], to create an ensemble of points distributed according to the PDF (8). Once we had the ensembles, we calculated mean values of the parameters and their standard errors for each group: vehicle, SB10, and SB30, and used two-sample z-test to estimate statistical significance of the differences in parameter values between the groups. We assumed that p-value < 0.05 means that SB has a statistically-significant effect on the corresponding parameter of the model.

## Results

### SB-334867 suppresses stress-induced hyperthermia

**Fig 2:**
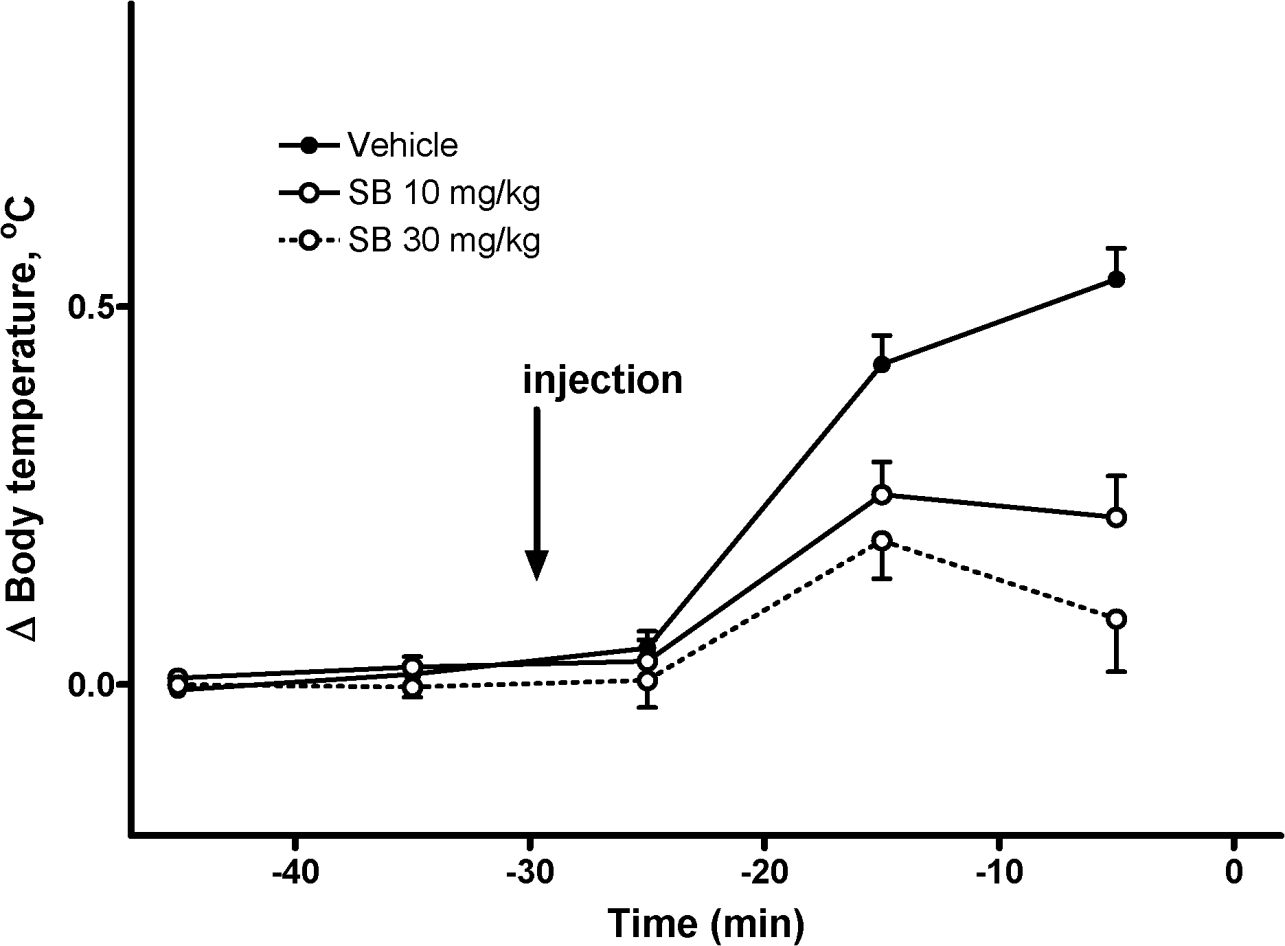
Effect of SB-334867 on stress-induced body temperature increase associated with i.p. injections (n = 24–26 per group). Black circles, solid line—pooled vehicle data; open circles, solid line—SB 10 mg/kg; open circles, dashed line—SB 30 mg/kg. Injection was performed at *t* = –30 min.

The first intraperitoneal injections evoked increases in the body temperature, which tended to return to baseline before the second injection in some groups. We have pooled the responses to the first injection in all groups before administration of Meth according to pretreatment to make four groups (two doses of SB and two vehicles for each dose of SB). However, there was no difference between responses to different vehicles, so data for both vehicles were pooled together as a single group. This reduced the number of groups to three. The increase of body temperature in response to the first injection was statistically significantly attenuated by both doses of SB (Fig 2), whereas there was no statistically significant difference between the two doses of SB (p>0.05).

### Effects of SB-334867 on temperature responses to Meth

The effects of Meth on body temperature were not different between the two vehicles used for the different doses of SB. These effects had complex dose-dependence, which has previously been described [29, 30, 38, 39]. In short, low (1 mg/kg) and high doses of Meth (10 mg/kg) evoked virtually immediate response while response to intermediate dose (5 mg/kg) was delayed. Peak hyperthermia after low and intermediate doses was similar (38.2 ± 0.1°C vs 38.2±0.1°C) but occurred at different moments (76±11 min vs 226±15 min after injection). The highest dose of Meth evoked a robust hyperthermia with temperatures reaching 39.5±0.2°C within 90 min of administration with the temperature subsequently declining from 90 to 150 min, followed by a plateau until the end of the recording period (up to 200 min). Injections of saline evoked slight increases of body temperature, which were short-lived: temperature returned to baseline within 60 min.

**Fig 3:**
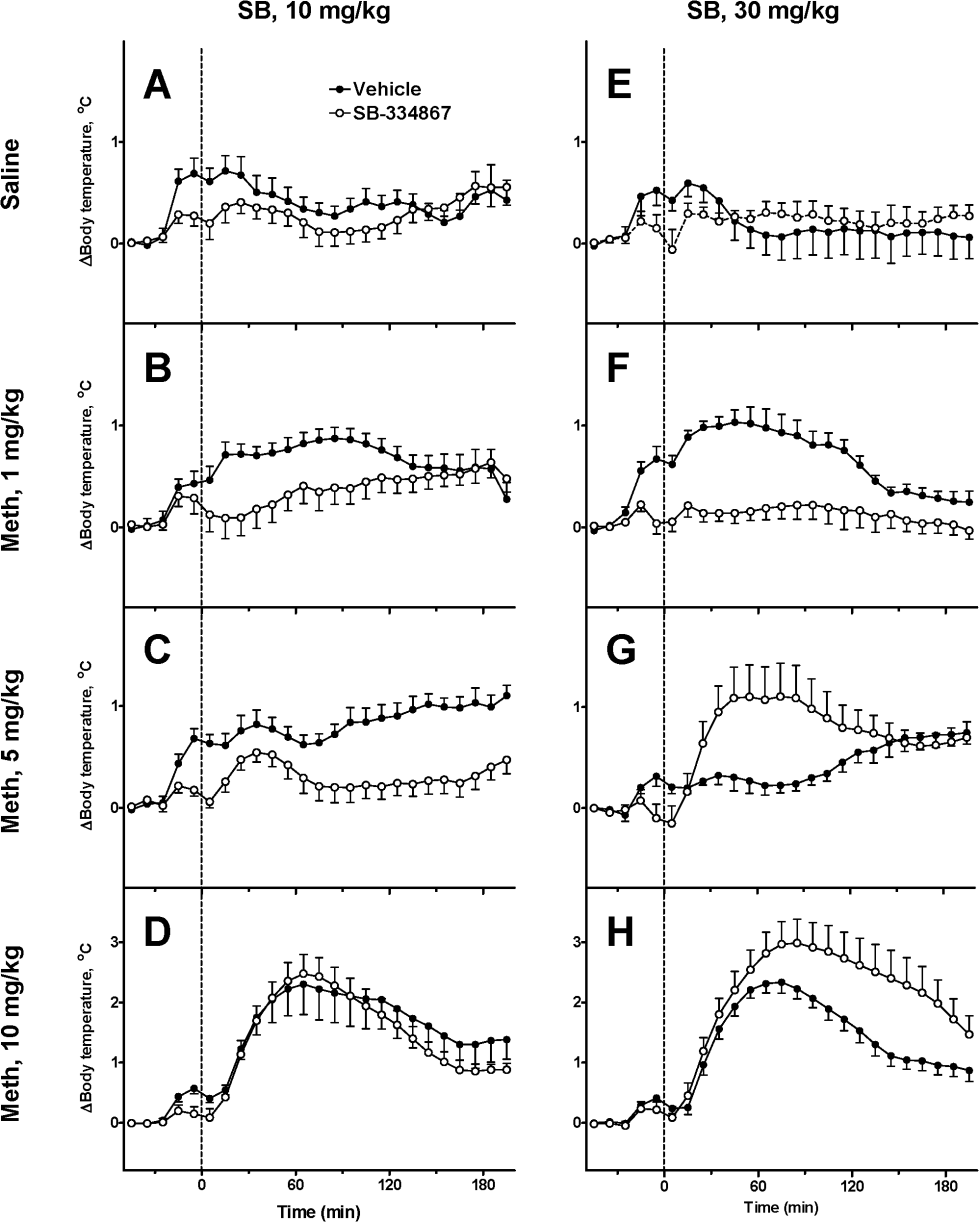
Changes in the body temperature evoked by methamphetamine after two doses of SB-334867. Meth is injected at *t* = 0 min (dashed lines). SB is injected 30 min prior to Meth. **A-D**: SB 10 mg/kg; **E-H**: SB 30 mg/kg. Each plot contains responses to the dose of Meth shown on the left: 0 (Saline), 1, 5 or 10 mg/kg after injection of either vehicle (black circles) or SB (open circles).

The responses to the same doses of Meth after SB are shown in Fig 3. After the low dose of Meth (1 mg/kg), rats treated with SB had lower body temperature than rats treated with vehicle during 3 h after administration of Meth (Fig 3B and 3F). However, this difference did not at any time reach statistical significance in the experiment with the lower dose of SB (10 mg/kg, Fig 3B). However, the suppression of the response to the low dose of Meth became statistically significant after the injection of higher dose of the antagonist (30 mg/kg, Fig 3F).

Next, after an intermediate dose of Meth (5 mg/kg) temperature transiently increased but remained lower than temperatures in the vehicle group. Furthermore, this dose of SB significantly attenuated thermogenic response after 70–90 min. In contrast, after the higher dose of SB (30 mg/kg) intermediate dose of Meth evoked a rapid hyperthermic response (Fig 3G) such that core temperature rose statistically significantly above the vehicle group. Qualitatively this response resembles a temperature response to a high dose of Meth in the vehicle group (Fig 3D).

Finally, the response to a high dose of Meth (10 mg/kg) was not modified by the lower dose of SB (Fig 3D) whereas the higher dose of SB significantly increased and prolonged the response to the high dose of Meth with the difference progressively increasing for over 120 min after the administration of Meth.

### Effects of SB on estimates of the model parameters

**Fig 4:**
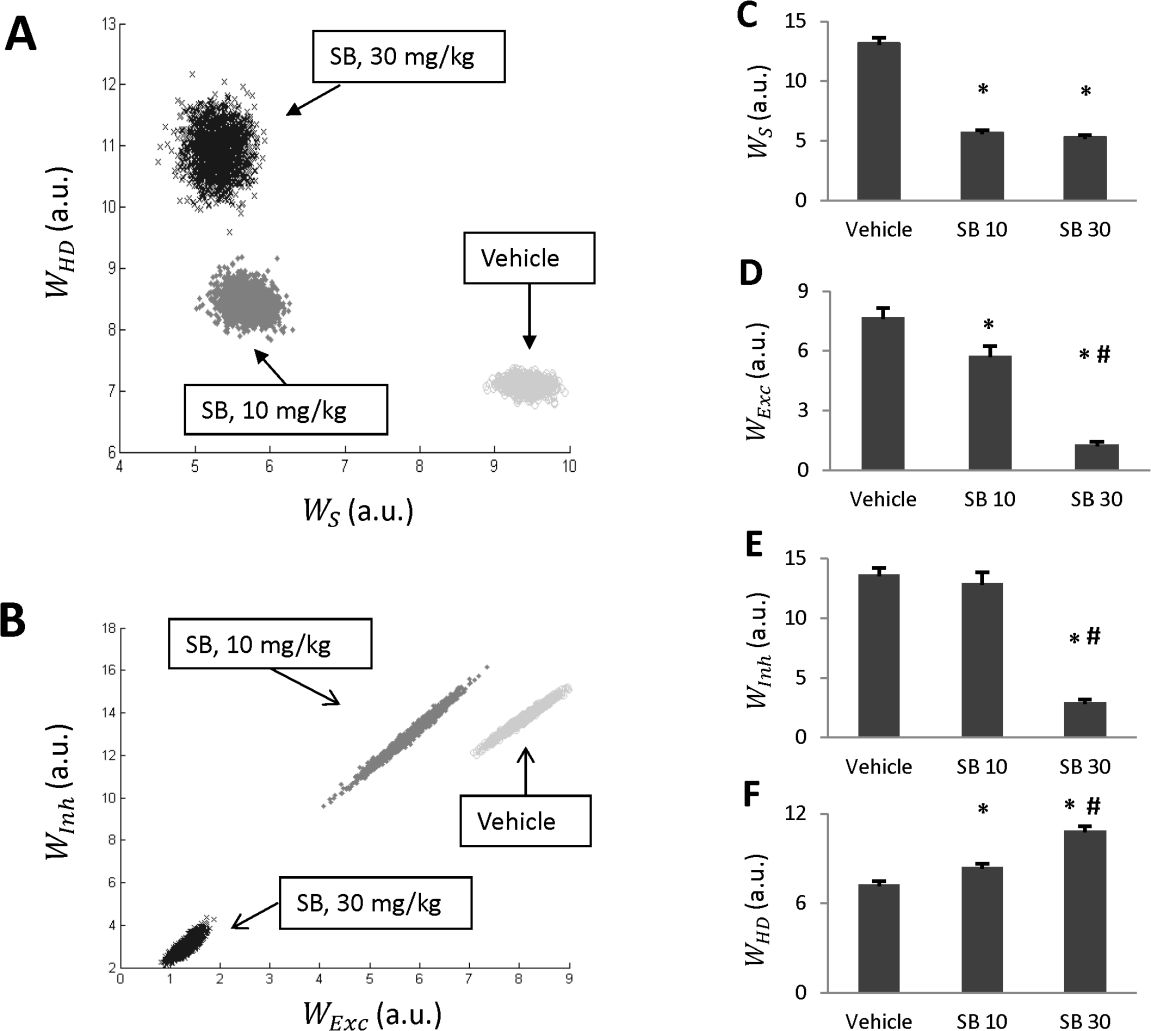
Parameter estimates generated by Monte-Carlo simulations. A-B: Statistical ensembles of parameters (amplitude of stress, and Meth-sensitivities of three Meth-dependent populations) projected onto (*w*_*S*_, *w*_*HD*_)-plane (A) and (*w*_*Exc*_, *w*_*Inh*_)-plane (B) for each of the three doses of SB (vehicle, 10 and 30 mg/kg). C-F: Individual parameters for each dose of SB. * denotes statistically significant difference from vehicle (SB 0, p<0.05); #—between SB 10 and SB 30 (p<0.05).

In this study we used the Metropolis algorithm to implement a Markov Chain Monte Carlo approach to estimate the posterior distribution of model parameters. Specifically, this algorithm generates a multidimensional cloud of points in the parameter space distributed according to the PDF (formula 8 in Methods) for a given experimental series {*T*_*i*_}. Four parameters (*w*_*S*_, *w*_*Exc*_, *w*_*Inh*_, *w*_*HD*_) have been sampled for three different doses of SB. We demonstrate the corresponding clouds in projections onto (*w*_*Exc*_, *w*_*Inh*_) and onto (*w*_*S*_, *w*_*HD*_)-planes shown in Fig 4A and 4B respectively. Each panel contains three clouds labeled “Vehicle”, “SB, 10 mg/kg” and “SB, 30 mg/kg”. These clouds are well separated implying that the differences in parameter values are statistically significant.

Statistical analysis confirms that antagonism of orexin receptors leads to statistically significant changes in estimates of the model parameters. Particularly, the stress amplitude *w*_*S*_ is reduced by administration of the orexin antagonist (Fig 4C), although, there was no significant difference in *w*_*S*_ estimates between 10 and 30 mg/kg of the antagonist. Also, parameters of sensitivity to Meth of both excitatory and inhibitory drives, *w*_*Exc*_, *w*_*Inh*_, were decreased by administration of SB (Fig 4D and 4E). The suppression was dose-dependent. The lower dose of the antagonist significantly decreased *w*_*Exc*_, but the effect was virtually absent for *w*_*Inh*_. However, the higher dose of SB was much more efficient in suppressing the Meth sensitivity of both the inhibitory and excitatory drives. Finally, the Meth sensitivity of HD node was progressively increased with increasing dose of SB (Fig 4F) implying an ability of this population to be activated by progressively lower doses of Meth.

### Effects of SB on the network components

**Fig 5:**
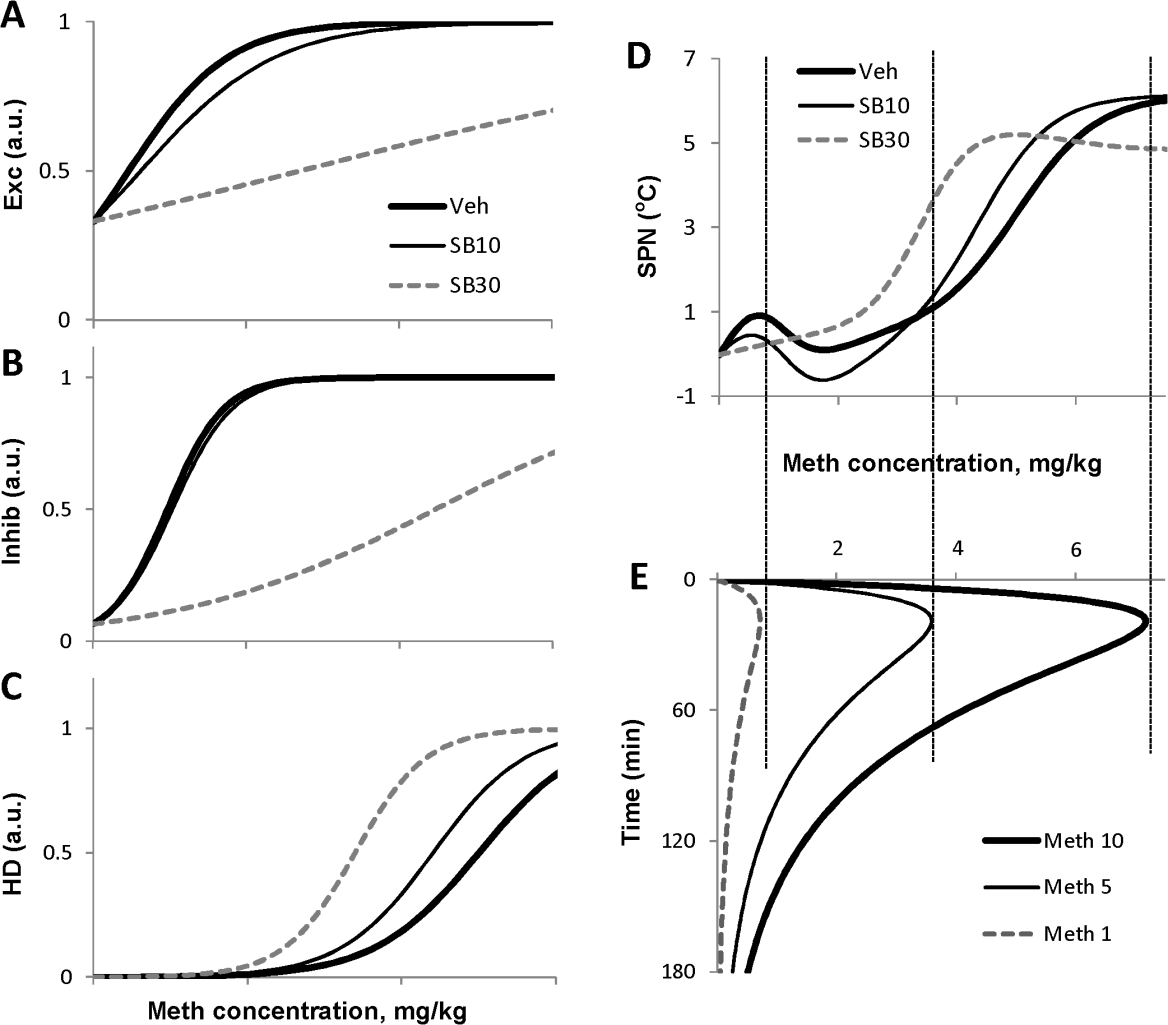
Activation curves of nodes of the model compared with pharmacokinetics of Meth. A-D: Activity of the network nodes as functions of Meth concentration in the blood after three doses of SB-334867: 0 (Veh), 10 and 30 mg/kg using best fit parameters. E: Time courses of the Meth concentration after different doses of Meth (1, 5 and 10 mg/kg) as generated by the model.

Of particular interest is how SB-evoked changes in the parameters of the model affect activation profiles of Meth-sensitive network components. Activities of *Exc*, *Inhib* and *HD* as functions of the blood concentration of Meth are shown at Fig 5A, 5B and 5C. SB downgrades the slope of the dose-dependence curve in case of *Exc* and *Inhib*, which signifies desensitization of these nodes (more Meth is required to evoke same response). The effect of SB on *Exc* is significant for both doses of antagonist and increases with dose. In contrast, an effect of the lower dose of SB on *Inhib* is subtle. Unlike two other Meth sensitive populations, *HD* gains higher sensitivity to Meth when SB is present as its dose-dependence curve shifts to the left. Therefore, lower concentrations of Meth are able to activate this neuronal population.

Superposition of inputs from these populations results in complex modification of *SPN* activity which ultimately controls the thermal response. The dose-dependence of *SPN* activity is shown in the Fig 5D, which is aligned with simulated pharmacokinetic profile of Meth after various doses of Meth used in this study (Fig 5E). The low dose of Meth (1 mg/kg, Meth-1 in. 5E) does not evoke any significant response when either of SB doses is present.

The intermediate Meth dose (5 mg/kg, Meth-5 in Fig 5E) is sufficient to elicit strong thermogenesis after the higher dose of SB (30 mg/kg, SB30 in Fig 5D) but not after the lower dose of the antagonist (10 mg/kg, SB10 in Fig 5D). 10 mg/kg of SB desensitizes *Exc*, barely affects *Inhib*, and sensitizes *HD*. Together these changes leave the activity of *SPN* at the peak Meth concentration caused by the injection of 5 mg/kg of Meth almost unaltered (Fig 5D and 5E). In contrast, 30 mg/kg of SB sensitize HD a little more (Fig 5C), but more importantly this dose substantially desensitizes the *Inhib* population (Fig 5B) leading to disinhibition of *SPN* activity (compare SB10 and SB30 in Fig 5D).

As mentioned SB induces sensitization of *HD* population to Meth (Figs 4F and 5C) which results in longer activation of the *HD* component by the same Meth concentration profile (Meth-10 in Fig 5E) due to progressively lower *HD* activation threshold (compare Veh, SB10 and SB30 in Fig 5D).

### Effects of SB on the temperature responses to Meth explained

#### Control responses to Meth

**Fig 6:**
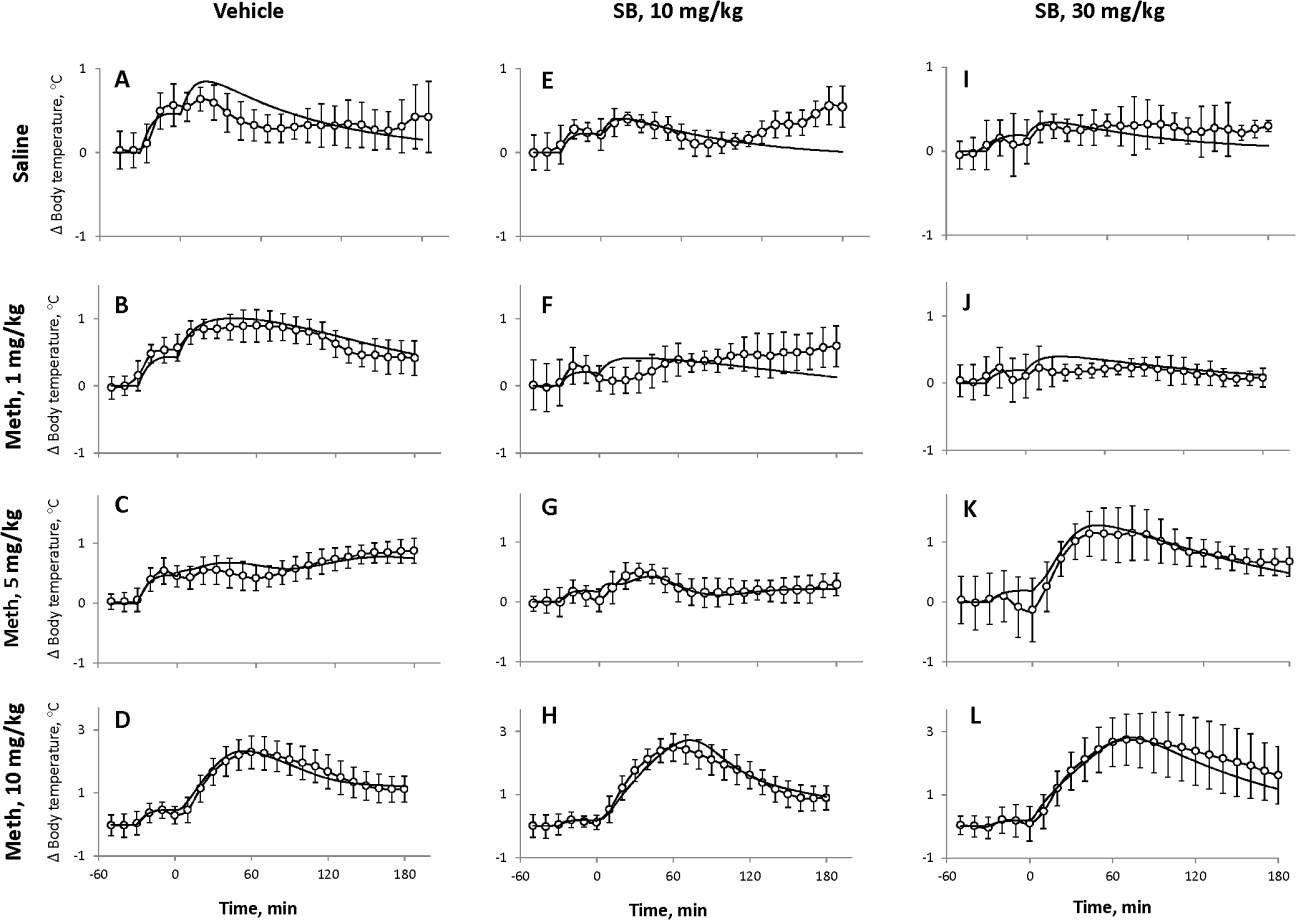
Comparison of experimental data and model responses. Experimental data shown by open circles (average temperature); error bars represent standard deviations over a group of rats. Model responses using best fit parameters are shown as a thick lines. SB vehicles for both doses were pooled into a single group. Columns of graphs correspond to different doses of the antagonist (A-D: Vehicle, E-H: 10 mg/kg and I-K: 30 mg/kg as marked at the top), rows of graphs correspond to different doses of Meth (Saline, 1, 5 and 10 mg/kg, as marked at the left).

Without SB and Meth, injections of the vehicle and saline cause stress which activates *Exc* (see Fig 1). This activation results in two early “peaks” of temperature in the beginning due to two injections separated by 30 min, followed by a decline to baseline (Fig 6A).

For 1 mg/kg of Meth, stress and Meth together activate *Exc* far greater than *Inhib* resulting in higher and longer maximum temperature after 1 mg/kg than after saline, however, the gradual decline afterwards was similar (Fig 6B).

Following the vehicle, the intermediate dose of Meth (5 mg/kg) activates both *Exc* and *Inhib* to their full extent effectively cancelling each other, and the second “stress bump” almost completely disappears. Accordingly, the body temperature does not react until after 60 min when the Meth concentration falls below the activation threshold of *Inhib*. This explains the plateau and gradual increase in temperature in the late phase (Fig 6C).

Finally, after 10 mg/kg of Meth at the early stage the activation levels of *Exc* and *Inhib* are saturated and negating each other. *HD* component is activated which results in an immediate robust temperature increase (Fig 6D). After approximately 70 min the Meth concentration falls below *HD*’s activation threshold, and the temperature starts declining. Later on, in about 120 min, Meth decreases enough to deactivate *Inhib* and, thus, to disinhibit *Exc* which temporarily slows the temperature drop or even initiates a secondary peak (not seen in Fig 6D).

For more details about temperature responses to Meth alone see [30].

#### Responses to Meth after SB

Administration of SB itself results in a spike in temperature due to stress caused by the injection which is partially suppressed by the drug (see Fig 2). The second bump on the temperature curve in Fig 6E corresponds to the injection of saline. The suppression of the responses to stress does not increase significantly with increasing dose of the orexin antagonist (compare Fig 6E and 6I).

After administration of SB the temperature response to the low dose of Meth does not exceed that of animals injected with saline (compare Fig 6F and 6J with Fig 6E and 6I) suggesting that the temperature responses to the low dose of Meth are eliminated (see Fig 5D).

At the intermediate dose of Meth (5 mg/kg) the temperature responses are noticeably and differentially altered by 10 and 30 mg/kg of SB (Fig 6C, 6G and 6K). After 10 mg/kg of SB the temperature curve loses the late ascending phase present in Fig 5C. This modification is concerned with SB-evoked desensitization of *Exc* to Meth (see Figs 4D, 5A and 5D). After the higher dose of SB (30 mg/kg) there appears strong immediate hyperthermia (Fig 6K) explained by sensitization of *HD* and desensitization of *Inhib* (Fig 5B, 5C and 5D).

Finally, after 10 mg/kg of Meth, both *Exc* and *Inhib* are activated to some extent by overcoming the blockade, but activation of *HD* overshadows the activity of the two, producing progressively larger temperature increase with increasing dose of SB (Fig 6D, 6H and 6L). This augmented hyperthermia as well as its longer duration are also mediated by SB-induced sensitization of the *HD* node and desensitization of *Inhib* to Meth (see Fig 5).

#### Mechanism of HD sensitization

Orexin is an excitatory neurotransmitter [40]. Accordingly, a blockade of orexin receptors is expected to reduce the activity of the postsynaptic neurons in presence of orexinergic synaptic inputs. From this perspective, the desensitization of *Exc* and *Inhib* populations by the orexin antagonist allows for a straightforward interpretation. Specifically, we can assume that these two nodes receive orexinergic projections from Meth-activated neurons, and, hence, these inputs are suppressed by the orexin receptor antagonism (see Fig 2).

**Fig 7:**
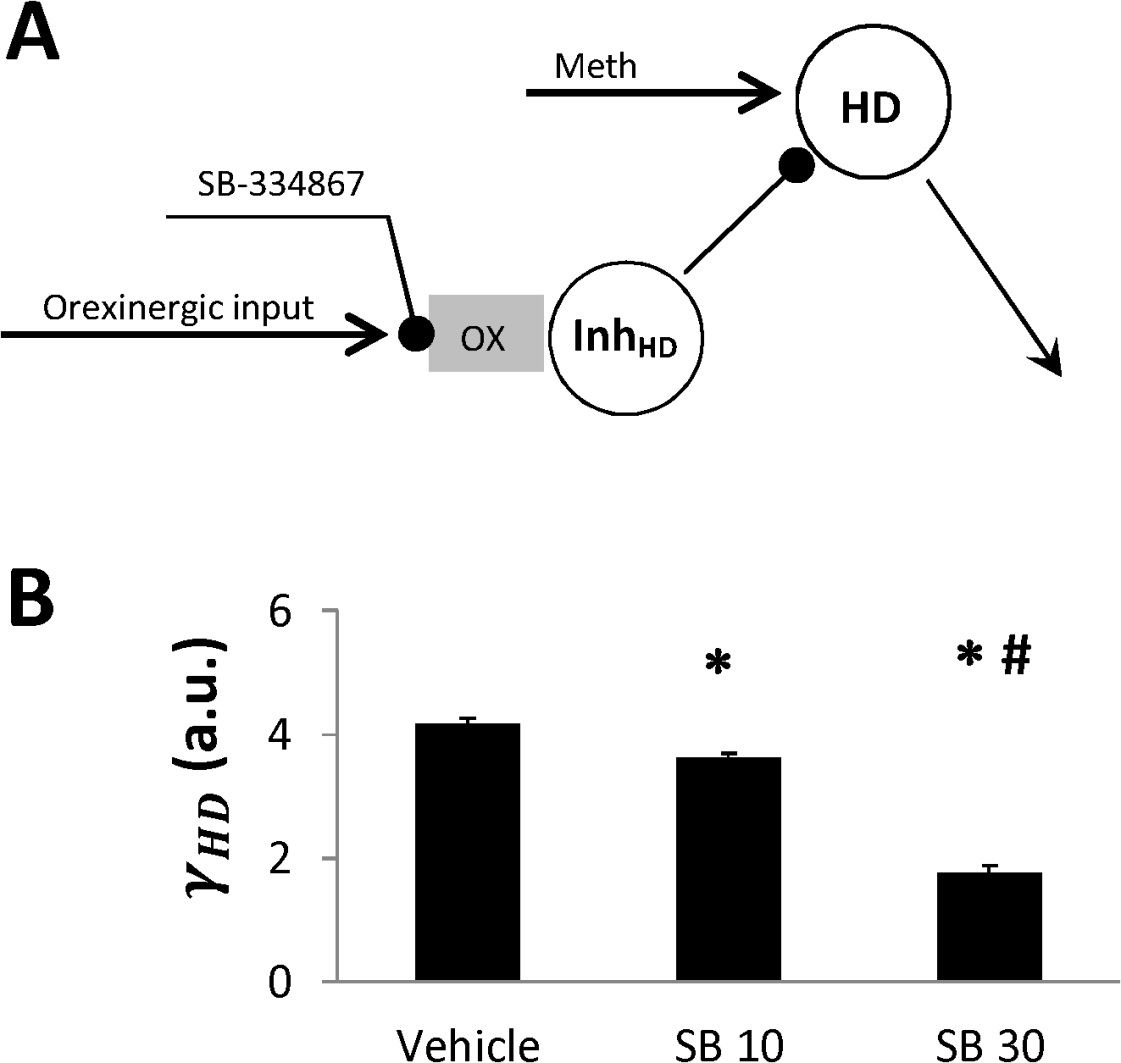
Interpretation of HD sensitization as a change of inhibitory tone to the HD. A: The mechanism of HD sensitization by the orexin receptor antagonist. SB suppresses activation of the inhibitory population Inh_HD_ by orexinergic input which disinhibits HD and thus lowers its activation threshold. B: Effect of SB on the basal inhibitory tone to HD population *γ*_*HD*_ when it is used as an SB-affected parameter instead of *w*_*HD*_. *—the estimate is statistically significantly different from the one for Vehicle (SB-0, p<0.05); #—the estimates are statistically significantly different between SB 10 and SB 30 (p<0.05). See Results for more details.

In the model presented in Fig 1 we assumed that SB affects the parameters of sensitivity of Meth-dependent populations *w*_*Exc*_, *w*_*Inh*_. This worked out very well to reproduce the data and is consistent with orexin excitatory action. In contrast, the *HD* population gets sensitized by the antagonist, which contradicts to the excitatory influence of orexin. Therefore, we assume that Meth-activated excitatory input, which this node receives, is not mediated by orexin. Instead, *HD* node may receive strong inhibition from another neural population whose activity is controlled by orexin receptors. In the presence of the orexin antagonist the activity of this inhibitory population gets reduced which in turn disinhibits *HD* (Fig 7A).Therefore, progressive sensitization of *HD* (Fig 4F), which was described above by an increase in *w*_*HD*_, is more likely a result of *HD* disinhibition as illustrated in Fig 7A.

The orexinergic drive in Fig 7A can be Meth-dependent or Meth-independent. In the former case the net Meth-dependent input to HD will be defined by the difference of Meth-induced excitation and inhibition. In presence of SB, the inhibition is reduced which results in the increased HD sensitivity to Meth characterized by the parameter *w*_*HD*_. In the latter case, the orexin antagonist does not affect the Meth-dependent input directly. It rather alters the basal excitability of the HD population defined by the orexin-dependent tonic inhibition it receives. Accordingly, a more biologically correct way to describe this is to alter *γ*_*HD*_ for different doses of SB, rather than *w*_*HD*_.

*γ*_*HD*_ estimates obtained by Monte Carlo simulations using (*w*_*S*_, *w*_*Exc*_, *w*_*Inh*_, *γ*_*HD*_) as a set of SB-affected parameters are shown in Fig 7B. As expected, this parameter progressively declines with increasing SB dose which leads to changes in *HD* activation curve almost indistinguishable from shown in Fig 5C. Obviously, the likelihood for these two models is almost the same, and hence they are equally probable from Bayesian standpoint.

To summarize, the sensitization of *HD* component of the network is concerned with its disinhibition. Our experimental data does not allow for a conclusion if this inhibition is Meth-activated or not.

### Effect of inhibition failure

**Fig 8:**
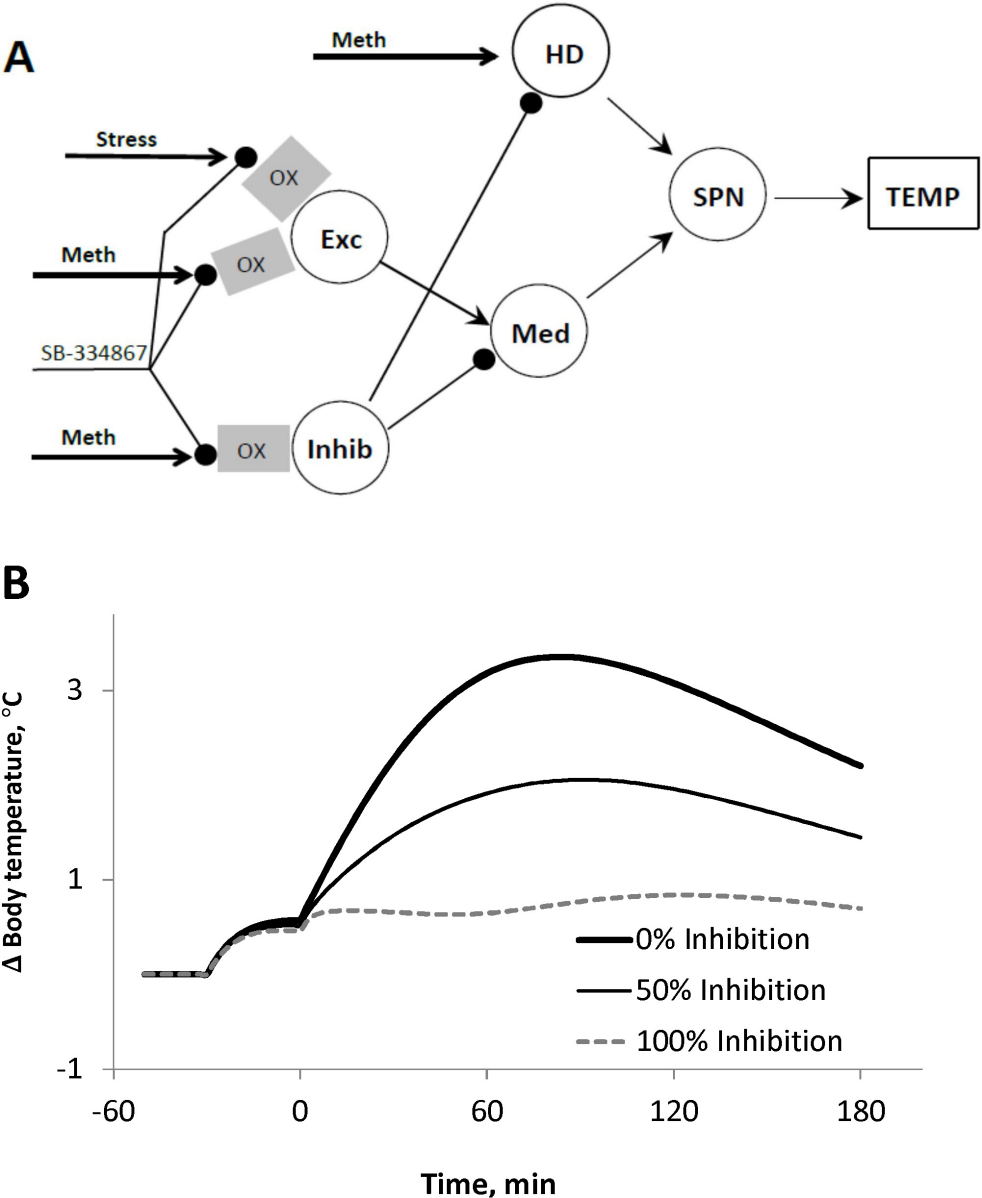
Reduced model and effect of inhibitory failure on the response. A. The extended model schematic in which the inhibitory tone to HD originates from Inhib population (see explanations in the Results). B: Effect of inhibitory failure in the modified model on the response to 3 mg/kg of Meth (see details in the Results).

The inhibitory control of *HD* population described above may originate from the *Inhib* node of the circuitry (see Fig 1). In this case we can synthesize the network architecture by combining the schematics from Figs 1A and 7A as shown in Fig 8A. We implemented a corresponding mathematical model and found that it fitted our experimental data with comparable precision (not shown) and hence had similar likelihood.

This model has a very important implication for the role of inhibition in this system. Indeed, both excitatory components, *Exc* and *HD*, appear to be controlled by the inhibitory drive whose failure may have dramatic consequences. Our simulations demonstrate the consequences of such failure. Fig 8B demonstrates how temperature responses to Meth change if the maximal activity of *Inhib* population is bounded. In our simulations we used a relatively low dose (3 mg/kg) which does not produce major response under baseline conditions (see unmodified 100% inhibition in Fig 8B). If the maximal activity of the *Inhib* population was decreased by two times (50% inhibition), the body temperature rose by almost 2 degrees. When *Inhib* was completely silenced, the body temperature reached life-threatening levels at almost 40°C (0% inhibition in Fig 8B).

## Discussion

This study builds upon previously published mathematical models [30], which were based on the premise that body temperature response to Meth is dependent upon the interaction between two neural components: excitatory and inhibitory drives. The difference in strength between these two signals ultimately determines the dynamics of body temperature.

The injections of SB (0 mg/kg, 10 mg/kg, and 30 mg/kg) differentially modify temperature responses to low, intermediate and high doses of Meth (0 mg/kg, 1 mg/kg, 5 mg/kg, and 10 mg/kg). Previously it was reported that 10 mg/kg of SB suppressed the temperature response to stress of injection and also late hyperthermia induced by the intermediate (5 mg/kg) dose of Meth [29]. The limited conclusiveness of that study was concerned with the fact that SB-related effects on the temperature response in the early phase (t < 60 min) were impossible to separate from stress-induced temperature fluctuations resulting from two intraperitoneal injections.

In this study using mathematical modeling we were able to reliably decompose the effect of SB into stress- and Meth-related components, and thus provide a straight-forward mechanistic explanation for the intricate alterations of the temperature responses to different doses of Meth induced by orexin receptor antagonism.

### Two excitatory components in temperature responses to Meth

In our previous publication [30], where we suggested the circuitry shown in Fig 1 to explain the complexity of the temperature responses to Meth, we also considered an alternative model where the excitatory populations, *Exc* and *HD*, were combined into a single excitatory node. The data used in that study failed to emphasize the plausibility of either architecture.

In the present study we have shown that two distinct excitatory populations are necessary to explain differential effects of the orexin receptor antagonist on the responses to low and high doses of Meth. Specifically, activation of the first excitatory population (*Exc* in Fig 8A) by low doses of Meth is mediated by orexinergic neurotransmission, while the direct Meth-dependent input to the second population (*HD*) does not rely on orexin receptor activation.

The details of the network architecture shown in Figs 7A and 8A clarify the nature of high activation threshold of the *HD* component which is yet another distinctive feature of the second excitatory population in the network. The lack of response of this population to low doses of Meth is not concerned with its lower sensitivity, but rather defined by a tight inhibitory control originated either from *Inhib* or some other inhibitory neuronal population(s). High doses of Meth are needed to overcome this inhibition. In either case the source of this inhibition is dependent upon orexinergic input. Orexin receptor antagonism suppresses the inhibition and thus lowers *HD* activation threshold which underlies SB-induced exaggeration of responses to high doses of Meth.

### Biological implications

The results of this work have several important implications to consider.

Amphetamines are known to activate orexin-containing neurons in the perifornical area and dorsomedial hypothalamus [20, 21]. From the phenomena observed in our experiments we conclude that this activation of orexinergic cells is required for temperature responses to Meth. Due to absence of hypothermia after administration of SB, we conclude that existing orexinergic projection to raphe pallidus [41] does not mediate temperature responses to Meth based on assumed locations of Excitatory and Medullary nodes [30]. In turn, the entire hypothalamus including dorsomedial hypothalamus contains orexin-containing fibers [41], which can mediate the above effects of Meth. However, this transmission can be not mono-, but polysynaptic (for example, perifornical area—ventral tegmental area—hypothalamus, or have even more connections).

The *Inhib* population in the network plays an extremely important role in counteracting hyperthermia. Previously, we have demonstrated that an imbalance of excitatory and inhibitory components can result in significant amplification of the response to relatively low doses of Meth [30]. The details of the circuitry proposed in this study emphasize the importance of the inhibitory component(s) even more. Simulations shown in Fig 8B demonstrate that a relatively low dose (3 mg/kg), which does not induce virtually any response in intact animals (see 100% inhibition in Fig 8B), can evoke profound hyperthermia typical for much higher doses (compare 0% inhibition in Fig 8B with Fig 6D). The impairment of the inhibitory control can not only allow excitation to pass through *Med*, but also lower the *HD* activation threshold so that this population gets activated by relatively low Meth doses.

Another implication of our results is that using orexin receptor blockade as a treatment for other ailments could cause life-threatening side effects. In particular, orexin antagonists were proposed to prevent or treat addictions to various drugs [42, 43]. An increase in excitability of the *HD* population in presence of SB highlights a potential danger of its use to treat drug abuse if the person uses the drug during treatment. Indeed, suppression of acute effects of amphetamines by the orexin antagonist may force an abuser to increase the dose which may cause life-threatening hyperthermia due to a dysfunction of the inhibitory population(s). Similarly, our model predicts that narcoleptic patients, whose condition is putatively caused by a lack of orexin [44], may be at higher risk of life-threatening overdose of amphetamine-like stimulants, which are usually prescribed to treat such patients [45].

Tupone, Madden (26) reported that administration of orexin into raphe pallidus evoked activation of thermogenesis in the BAT and increased body temperature in anesthetized rats. Surprisingly, local microinjection of SB into the same area also induced thermogenesis and hyperthermia [26]. Our analysis provides possible interpretation of this data as the network has both excitatory and inhibitory populations activated by orexin. Namely, activation of the excitatory drive by orexin can evoke thermogenic response, whereas a blockade of orexin receptors in the inhibitory population will remove the inhibitory tone which may result in a similar effect.

## Conclusions

In this study, using mathematical modeling, we mechanistically explained non-trivial alterations of temperature response to various doses of Meth at presence of the orexin receptor antagonist. Our model can be used to generate experimentally-testable predictions on the thermogenic effects of Meth when activity of orexin receptors is modified. The model has several essential features regarding the mechanisms of Meth-evoked hyperthermia and stress response.

- Both low dose of Meth and stress activate the excitatory component which is mediated by orexin receptors and hence can be suppressed by SB.
- The inhibitory component of the response to Meth is also mediated by orexin.
- Orexin antagonism disinhibits, and thus increases the sensitivity of the component normally activated by the high dose of Meth.
- Insufficient inhibitory drive can cause fatal hyperthermia after relatively low doses of Meth. This insufficiency can be provoked by a decreased orexinergic tone either due to deficiency of orexinergic transmission (narcolepsy) or after administration of orexin receptor antagonists (e.g. used for drug abuse treatments).

## Supporting Information

S1 Dataset Body temperature changes induced by methamphetamine in rats pretreated with different doses of SB-334867. (XLSX)
